# Clivopalate angle: a new diagnostic method for basilar invagination at magnetic resonance imaging

**DOI:** 10.1007/s00330-018-5972-3

**Published:** 2019-02-08

**Authors:** Lichao Ma, Liuji Guo, Xiaodan Li, Jie Qin, Wenle He, Xiang Xiao, Lijun Lu, Yikai Xu, Yuankui Wu

**Affiliations:** Department of Medical Imaging, Nanfang Hospital, Southern Medical University, No. 1838 Guangzhou Avenue North, Guangzhou, Guangdong 510515 People’s Republic of China

**Keywords:** Atlanto-occipital joint, Platybasia, Cephalometry, Clivopalate, Magnetic resonance imaging

## Abstract

**Objectives:**

To investigate the diagnostic value of clivopalate angle (CPA) for basilar invagination (BI) at magnetic resonance imaging (MRI).

**Methods:**

In this retrospective case-control study, CPA, clivodens angle (CDA), and clivoaxial angle (CXA) were measured on midsagittal MR images from 112 patients with BI (22 men; mean age, 43.9 years ± 13.1 years; range, 21–79 years) and 200 control subjects (57 men; mean age, 47.1 years ± 13.3 years; range, 20–80 years). Intraclass correlation coefficient (ICC), linear regression, Mann-Whitney *U* test, binary logistic regression, and receiver operating characteristic (ROC) curve were used for statistical analysis.

**Results:**

Clivopalate angle showed better inter-observer agreement (ICC = 0.951) than CXA (0.867) or CDA (0.853). CPA significantly correlated with CXA (*R* = 0.811, *p* < 0.001) and CDA (*R* = 0.716, *p* < 0.001). Patients with BI had a significantly smaller CPA (45.9° ± 9.9°) than control subjects (61.9° ± 6.2°) (*p* < 0.001). With the optimal cutoff value of 53.5°, CPA had a sensitivity of 0.839 (94/112) and a specificity of 0.915 (183/200). The area under the ROC curve (AUC) was 0.937 (95% CI, 0.911–0.963) for CPA, which was similar to that of CXA (AUC, 0.957; 95% CI, 0.936–0.978) or CDA (AUC, 0.925; 95% CI, 0.892–0.957). The combination of CPA and CDA or CXA showed a higher diagnostic value than CDA or CXA alone.

**Conclusions:**

The diagnostic performance of CPA was similar to that of CXA or CDA, but CPA might be more reliable in evaluation of BI. CPA provided complementary information to CXA and CDA.

**Key Points:**

• *Clivopalate angle has a high diagnostic value for basilar invagination.*

• *Clivopalate angle demonstrates high inter-reader agreement than does clivoaxial angle or clivodens angle.*

• *Clivopalate angle provides complementary information to clivoaxial angle and clivodens angle.*

**Electronic supplementary material:**

The online version of this article (10.1007/s00330-018-5972-3) contains supplementary material, which is available to authorized users.

## Introduction

Basilar invagination (BI) is a common deformity of the craniovertebral junction (CVJ), characterized by flattening of the base of the skull and upward displacement (impression) of the basilar and condylar portions of the occipital bone and odontoid process, and is often accompanied by other complications such as other skeletal deformities, tonsillar herniation, and cervical syringomyelia [1–5]. Magnetic resonance imaging (MRI) plays a vital role in the diagnosis and management of BI.

Although a series of imaging indicators is available, clinicians still desire a more reliable MRI index that significantly correlates with clinical findings of cervical medullary syndrome and accurately monitors treatment efficacy in patients with BI [6, 7]. The distance from the odontoid tip to the Chamberlain line (CL), the line drawn from the posterior margin of the hard palate to the dorsal margin of the foramen magnum, is the most commonly used index for diagnosing BI [8, 9]. However, a major challenge of this method on MRI is accurate identification of the cortical bone of the odontoid tip, the posterior margin of the hard palate, and opisthion [8]. Furthermore, the anatomical variations of the odontoid process or changes in its position due to trauma may also significantly interfere with these measurements [10]. Many studies demonstrated that clivoaxial angle (CXA) or clivocanal angle, defined as the intersection of the Wackenheim clivus baseline with a line drawn along the posterior surface of the axis body and odontoid process, is a practical indicator for BI with high diagnostic value [11]. However, several factors, such as subjects’ cervical curvature, which might change significantly after surgery, as well as hyperostosis, may affect CXA measurement and thus compromise its diagnostic value for BI [6, 12]. Recently, Xu and Gong [13] proposed a new index developed from CXA, the clivodens angle (CDA), to overcome the above-stated shortcomings, whereas its diagnostic value for BI was similar to that of CXA.

We noticed that clivopalate angle (CPA), formed at the intersection of the Wackenheim line and a line along the hard palate plane, was different between patients with and without BI. Moreover, this angle is easy to measure because these two lines naturally exist and are clearly visible in the midsagittal MR image (refer to “Materials and methods” section). Thus, we hypothesized that CPA may be a useful indicator for BI. In this study, we measured CPA in 112 patients with BI and 200 control subjects to investigate its diagnostic value for BI at MRI.

## Materials and methods

This retrospective study received approval from the institutional review board and was performed with a waiver of informed consent.

### Study population

**Fig. 1:**
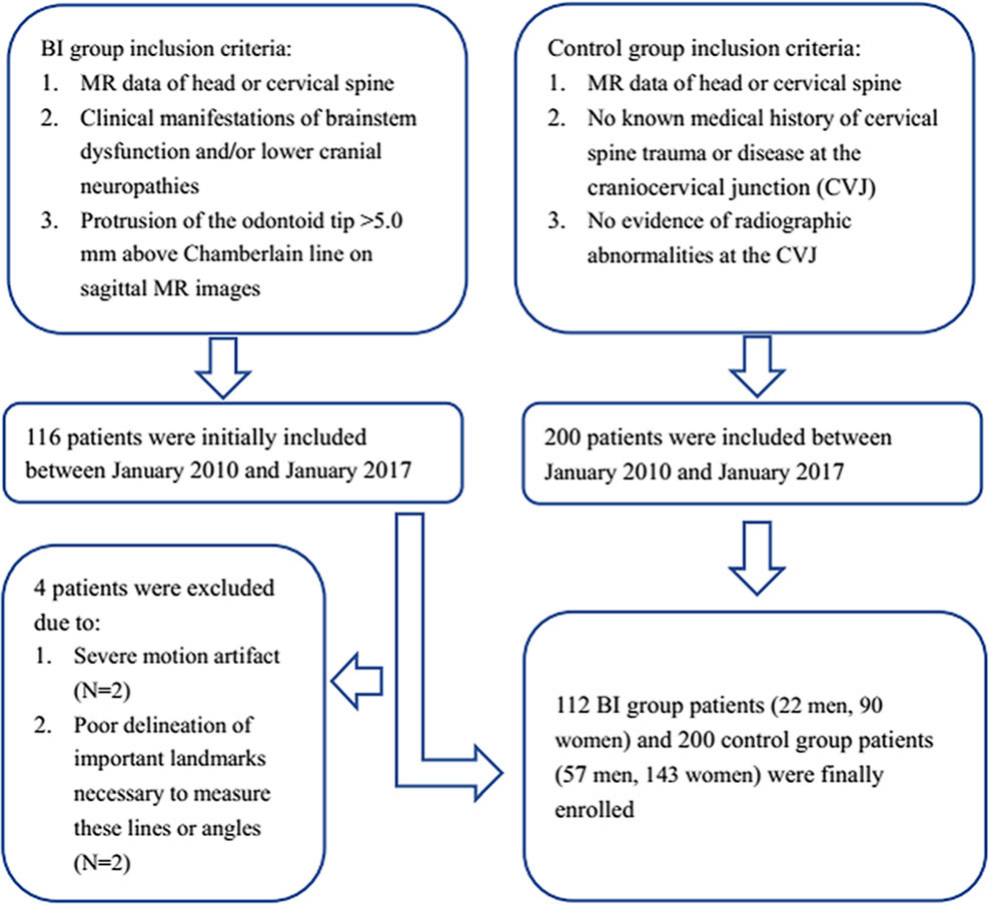
Flowchart of the study population

We used the picture archiving and communication system program to search our radiology database from January 1, 2010, to January 1, 2017, retrospectively (Fig. 1). One hundred and sixteen consecutive patients who presented with clinical manifestations of brainstem dysfunction and/or lower cranial neuropathies and with protrusion of the odontoid tip > 5.0 mm above the CL on sagittal MR images were recruited for the BI group. Four cases were excluded for severe motion artifact (two cases) or poor delineation of important landmarks necessary to measure these lines or angles (two cases). Eventually, 112 cases of BI patients (22 men and 90 women; mean age, 43.9 years ± 13.1 years; range, 21–79 years) with MRI data of the head (44 cases) or cervical spine (68 cases) were included in this study, with a mean duration of 5 years from symptom onset to clinic visit. Of these patients, 52 had cervical CT scan before undergoing MRI scan.

We enrolled 200 consecutive control subjects (57 men and 143 women; mean age, 47.1 years ± 13.3 years; range, 20–80 years) who had MRI data of the head (72 cases) or cervical spine (128 cases) with no known medical history of cervical spine trauma or disease at the CVJ. There was also no evidence of radiographic abnormalities, either on CT or MRI, at the CVJ in any of these control subjects. Patient information was anonymized and de-identified prior to analysis.

### Imaging protocol

Patients were examined on 3.0-T MRI scanners (Signa Excite, GE Healthcare; Achieva, Philips Healthcare) with the use of a head or head-neck coil. Sequences and parameters are summarized in supplementary materials (see Supplementary Table 1).

### Image analysis

**Fig. 2:**
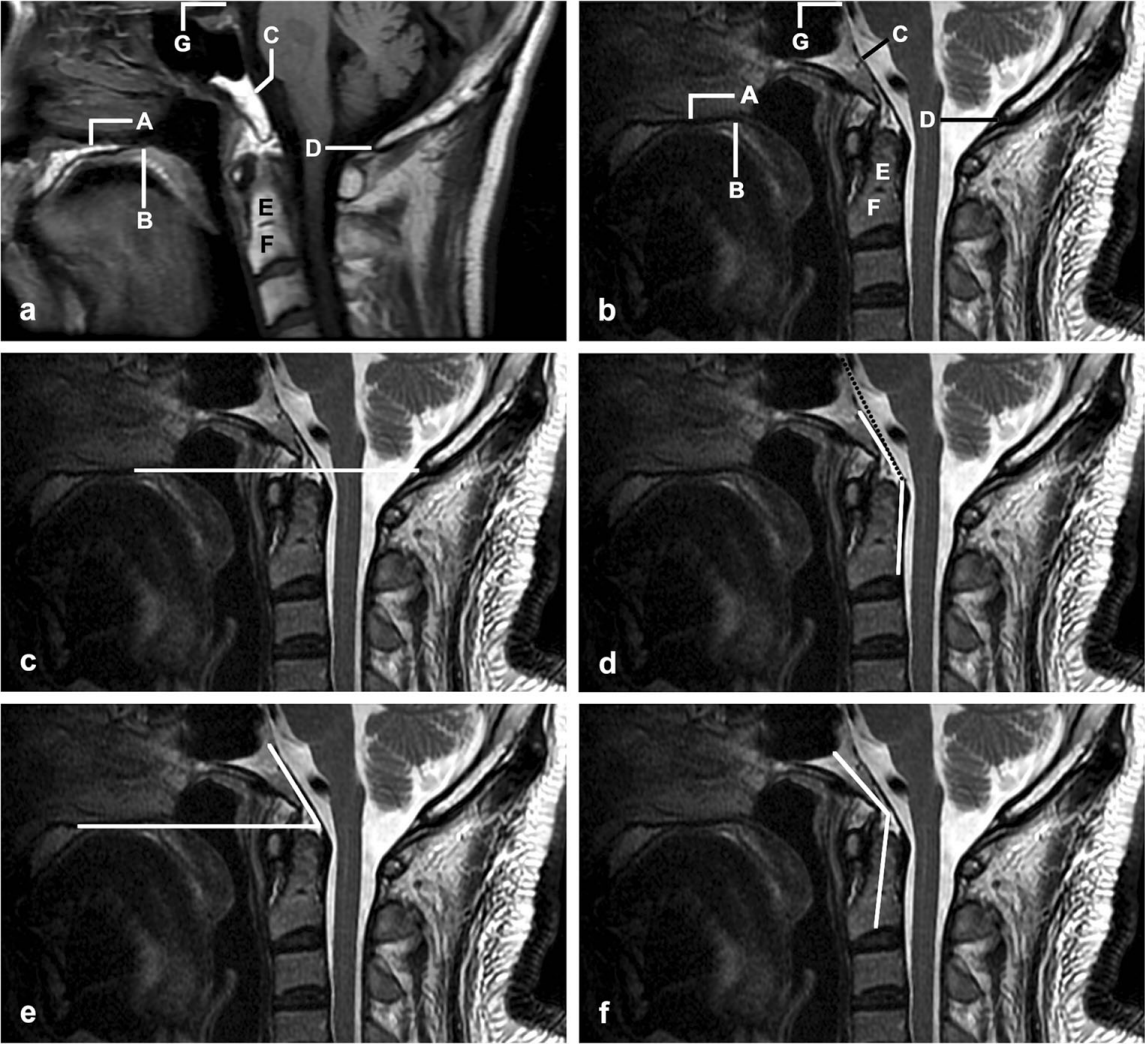
Lines and angles for the evaluation of basilar invagination. **a**, **b** Normal landmarks on midsagittal MRI T1-weighted and T2-weighted images, respectively. (A) Superior margin of the hard palate plane. (B) Dorsal margin of the hard palate. (C) Clivus. (D) Opisthion (dorsal margin of the foramen magnum). (E) Odontoid process. (F) Body of the axis. (G) Dorsum sellae. **c** Chamberlain’s line, the dorsal margin of the hard palate to the opisthion. **d** Clivoaxial angle (CXA), the angle formed at the junction of the newer Wackenheim line (white line, from the spheno-occipital synchondrosis to the top of the odontoid process) and the line along the posterior surface of the axis body and odontoid process. The dotted black line represents the traditional Wackenheim line from the top of the dorsum sellae to the top of the odontoid process. **e** Clivopalate angle (CPA), the angle formed at the junction of Wackenheim line and the hard palate line. **f** Clivodens angle (CDA), the angle formed at the intersection of a line along the long axis of the clivus and the one along the long axis of the odontoid process

CL, CPA, CDA, and CXA were measured in random order on midsagittal MR images of all 312 subjects by a radiologist (L.M. with 12 years of experience in CVJ image), blinded to subject demographics and clinical history. First, an attempt was made to identify the following anatomical landmarks: hard palate, clivus, dens, basion (the ventral margin of the foramen magnum), and opisthion (the dorsal margin of the foramen magnum) (Fig. 2a, b). Then, CL (Fig. 2c), CXA (Fig. 2d), CPA (Fig. 2e), and CDA (Fig. 2f) were measured as described previously or in the literature [6, 13]. The presence of other abnormalities of CVJ (i.e., atlas occipitalization, anterior atlantoaxial subluxation, Chiari malformation) and the morphologic abnormalities of dens and the hard palate was also evaluated according to the standard used in previously published studies [2, 11].

To assess the inter-observer reliability, an investigator (J.Q. with 3 years of experience in CVJ image) independently repeated the measurements of CPA, CXA, and CDA of all subjects.

### Statistical analysis

All descriptive and statistical analyses were performed using SPSS (version 20.0, IBM). The Kolmogorov-Smirnov test was used to evaluate the normality of continuous variables (age and angles). Sex and age were compared between the study and control groups by using chi-square test and independent-samples *t* test, respectively. The Mann-Whitney *U* test was utilized for comparing the mean values of CPA, CDA, and CXA of the two groups. Binary logistic regression and receiver operating characteristic (ROC) curve analyses were used to evaluate the diagnostic efficacy of CPA, CDA, and CXA when being assessed individually or in combinations. The differences between every two areas under the curve (AUCs) were compared according to the DeLong method using MedCalc (version 15.0, MedCalc software). Inter-observer reliability was evaluated with intraclass correlation coefficients (ICCs), which was interpreted as 0.41–0.60 representing moderate agreement, 0.61–0.80 representing substantial agreement, and 0.81–1.00 representing almost-perfect agreement. Linear regression was used to analyze the correlation between angles. All tests were two-sided with a 5% risk.

## Results

**Fig. 3:**
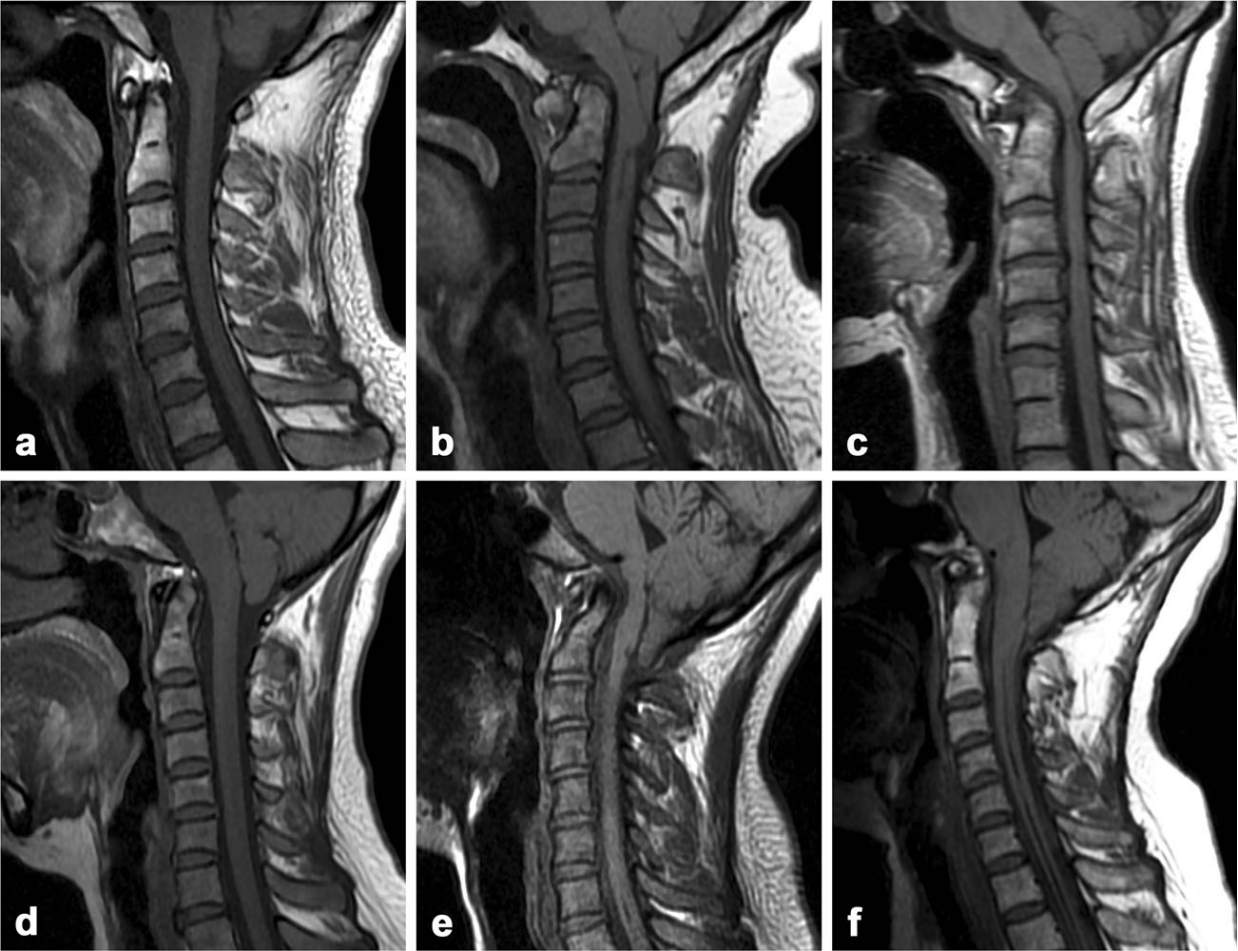
Morphological variations of the odontoid process. **a** Normal shape. **b** Anterior bow. **c** Antero-flexion. **d** Retro-flexion. **e** Serpentine type. **f** Prolonged type

No significant difference was observed between the study and control subjects in sex (*p* = 0.185) or age (*p* = 0.117). Chief complaints from patients in the study group are summarized in Table 1. Accompanied deformities in both groups are summarized in Table 2. The odontoid process showed five types of morphological variations (Fig. 3) in 50 cases in the BI group and in 18 cases in the control. No structural abnormality of the hard palate was identified in either group.

**Table 1:** Chief complaints from patients with basilar invagination

Symptom	Number (*N* = 112), *n* (%)
Headache	18 (16)
Dizziness	28 (25)
Numbness in face	2 (2)
Neck pain	52 (46)
Suboccipital pain	30 (27)
Unsteady gait	8 (7)
Hoarseness	6 (5)
Dysphagia	2 (2)
Numbness in limbs	24 (21)
Pain in limbs	24 (21)
Weakness in limbs	26 (23)
Limb muscle atrophy	8 (7)
Trauma history	2 (2)

**Table 2:** Accompanied deformities in the study and control groups

Deformity	Patients with BI (*N* = 112)	Control subjects (*N* = 200)
Atlantoaxial subluxation	70	0
Atlas occipitalization	74	0
C2–3 assimilation	5	0
Chiari malformation	66	0
Syringomyelia	64	0

*BI* basilar invagination

### Measurements from image analysis

The mean value of CL was 7.2 mm ± 3.8 mm (range, 5.8–14.0 mm) in the BI group, and − 1.1 mm ± 3.1 mm (range, − 7.6 mm to 8.0 mm) in the control group among whom five subjects had a CL value of greater than or equal to 5 mm (i.e., the false positive rate was 5/200). There was a perfect agreement between the two investigators in the measurement of all three angles (*p* < 0.001) with CPA exhibiting the highest inter-observer agreement (ICC values of 0.951, 0.867, and 0.853 for CPA, CXA, and CDA, respectively). Linear regression analysis showed a significant correlation between each pair of angles (*p* < 0.001) but a relatively low correlation between CPA and CXA (*R* = 0.811) and between CPA and CDA (*R* = 0.716) compared to the relatively high correlation between CXA and CDA (*R* = 0.903).

**Fig. 4:**
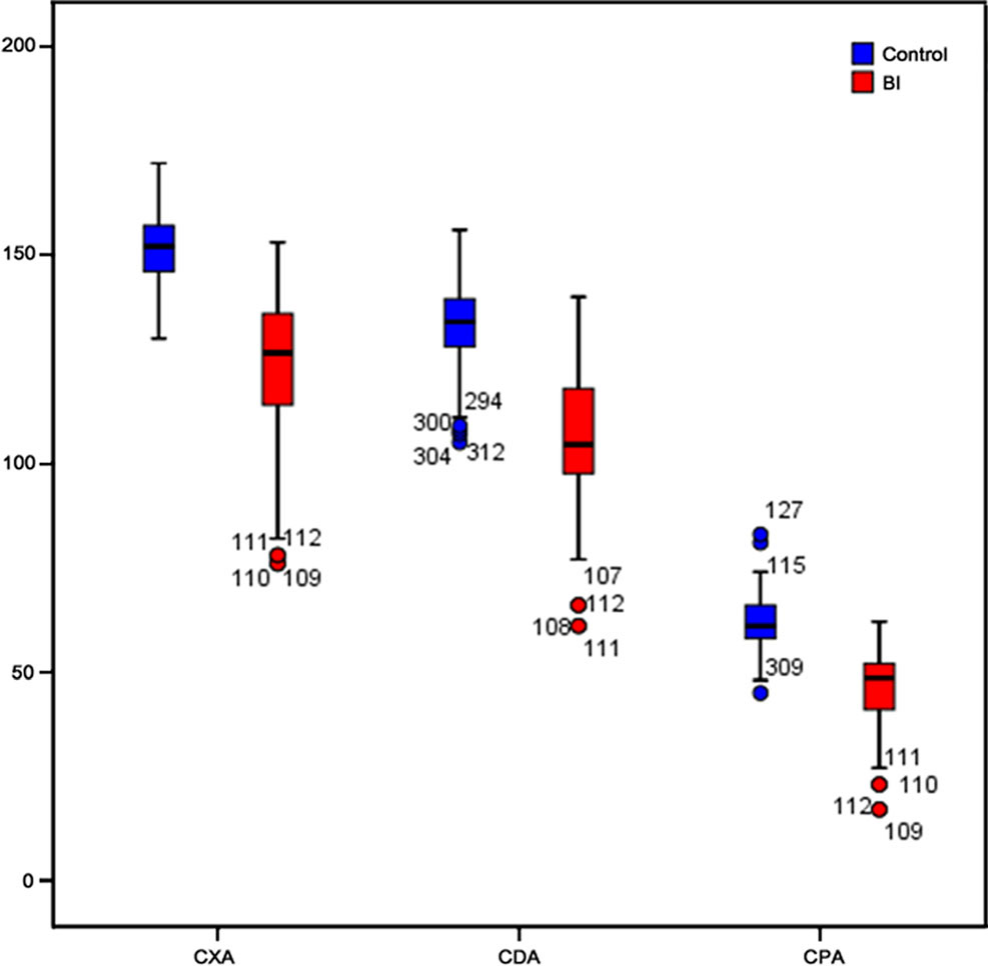
Three different angles for patients with basilar invagination. Compared with control subjects, patients with basilar invagination had a significantly smaller clivopalate angle (CPA), clivoaxial angle (CXA), and clivodens angle (CDA)

**Fig. 5:**
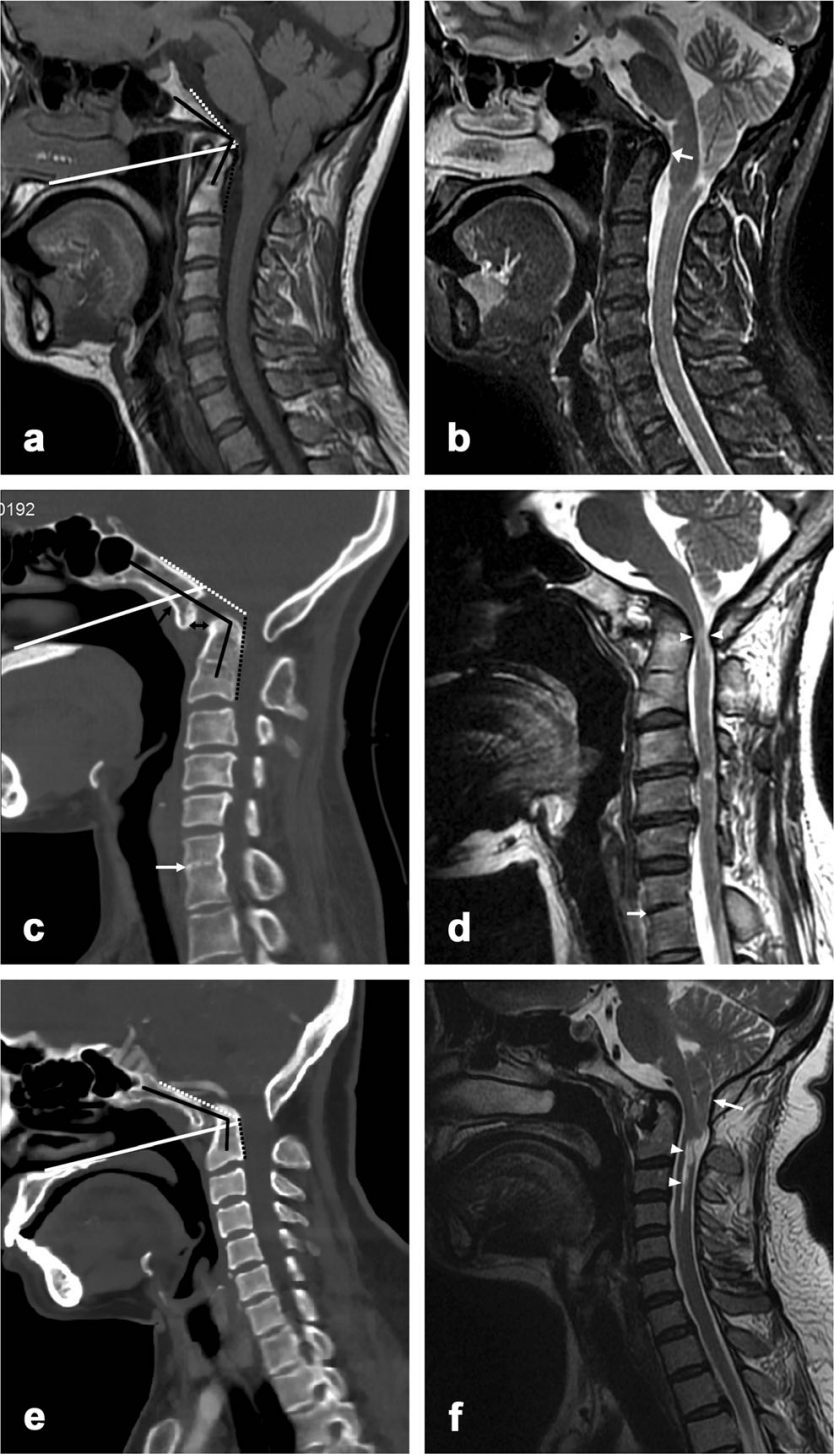
Three typical cases of basilar invagination. Midsagittal T1-weighted (**a**) and T2-weighted (**b**) images from a 45-year-old female demonstrating a significantly reduced clivoaxial angle (CXA; intersection of dotted white and black lines, 125°), clivodens angle (CDA; intersection of solid black lines, 100°), and clivopalate angle (CPA; intersection of the dotted white line and the solid white line, 52°), and a Chamberlain line (CL) value of 6 mm. Chiari II malformation and marked medullar compression (arrow) are also shown. Midsagittal CT (**c**) and T2-weighted (**d**) images from a 37-year-old female demonstrating abnormal CXA (118°), CDA (111°), and CPA (49°), and a CL value of about 6 mm. Atlas occipitalization (comma-shaped configuration, black arrow), atlantoaxial subluxation (double arrow), C6–7 assimilation (white arrow), as well as foramen magnum stenosis, cervical cord compression, and degeneration (white arrowhead) are also shown. Midsagittal CT (**e**) and T2-weighted (**f**) images from a 48-year-old female demonstrating abnormal CXA (122°), CDA (111°), and CPA (45°), and a CL value of 10 mm. Note the atlas occipitalization, atlantoaxial subluxation, syringomyelia (arrowhead), and Chiari I malformation (arrow)

The mean values of CPA, CXA, and CDA were 61.9° ± 6.2° (range, 45–83°; median, 61°), 151.7° ± 8.5° (range, 130–172°; median, 152°), and 133.4° ± 9.7° (range, 105–156°; median, 134°) in the control group, and 45.9° ± 9.9° (range, 17–62°; median, 48.5°), 123.5° ± 17.0° (range, 76–153°; median, 126.5°), and 106.7° ± 16.1° (range, 61–140°; median, 104.5°) in the study group, respectively. Compared with control subjects, patients with BI had significantly smaller CPA, CDA, and CXA (*p* < 0.001) (Fig. 4). Figure 5 shows three representative cases of BI within our study sample.

### Receiver operating characteristic analysis

**Fig. 6:**
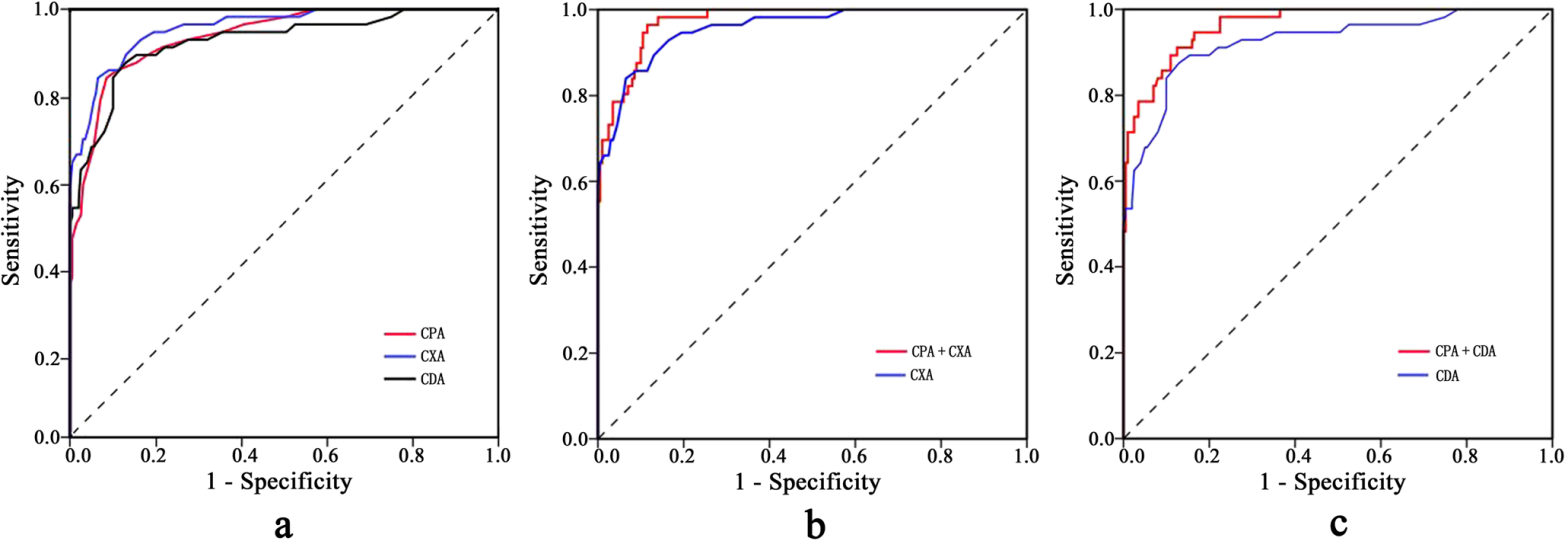
Receiver operating characteristic (ROC) curves of clivoaxial angle (CXA), clivodens angle (CDA), and clivopalate angle (CPA) as well as their combinations. **a** The areas under the curves (AUCs) for CPA, CXA, and CDA are 0.937, 0.957, and 0.925, respectively. **b**, **c** CPA shows the added value to both CXA and CDA

Table 3 summarizes the results of ROC analysis for CPA, CXA, and CDA. The optimal cutoff value was 53.5°, 138.5°, and 123.5° for CPA, CXA, and CDA, respectively. CPA demonstrated the sensitivity of 0.839 (94/112), specificity of 0.915 (183/200), and accuracy of 0.888 (277/312). The AUC of CPA was 0.937, which was not significantly different from that of CXA (AUC = 0.957, *p* = 0.183) or CDA (AUC = 0.925, *p* = 0.510) (see Supplementary Table 2). Figure 6a plots the ROC curve of each angle.

**Table 3:** Receiver operating characteristic analysis results of the three angles

Angle	AUC	Youden index	Cutoff value (°)	Se	Sp	Ac
CPA	0.937 (95% CI, 0.911–0.963)	0.754	53.5	0.839	0.915	0.888
CXA	0.957 (95% CI, 0.936–0.978)	0.774	138.5	0.839	0.935	0.901
CDA	0.925 (95% CI, 0.892–0.957)	0.745	123.5	0.875	0.870	0.872

*AUC* area under the curve, *CI* confidence interval, *Se* sensitivity, *Sp* specificity, *Ac* accuracy, *CPA* clivopalate angle, *CXA* clivoaxial angle, *CDA* clivodens angle

### Diagnostic value of different angles in combination

The combination of CXA and CDA did not improve the diagnostic performance beyond that of CXA (Supplementary Fig. 1), while the combination of CPA and CXA and that of CPA and CDA improved the diagnostic performance beyond that of CXA and CDA alone (see Table 4, Supplementary Table 2, and Fig. 6b, c), respectively. The combination of CPA and CXA showed the highest AUC value (0.973), sensitivity (0.964), and accuracy (0.913).

**Table 4:** The diagnostic value of combined angles for patients with basilar invagination

	AUC	Se	Sp	Ac
CPA + CXA	0.973 (95% CI, 0.959–0.987)	0.964	0.885	0.913
CPA + CDA	0.964 (95% CI, 0.947–0.981)	0.911	0.875	0.888
CXA + CDA	0.957 (95% CI, 0.936–0.978)	0.857	0.930	0.904

*AUC* area under the curve, *CI* confidence interval, *Se* sensitivity, *Sp* specificity, *Ac* accuracy, *CPA* clivopalate angle, *CXA* clivoaxial angle, *CDA* clivodens angle

## Discussion

Our present study demonstrated that measuring CPA on midsagittal MRI exhibited high inter-observer agreement. With an optimal cutoff value of 53.5°, CPA exhibited a high AUC value similar to that of CXA and CDA. Additionally, CPA provided complementary information to CXA or CDA for BI and improved the diagnostic performance.

The distance from the odontoid tip to the CL is the most widely used index for diagnosing BI [11, 14]. Generally, a CL value ≥ 5 mm is suggestive of BI [5, 12, 15, 16]. In contrast, using a CL value of 2.5 mm as the criterion may produce a relatively high false positive rate [13]. Thus, the criterion was set at 5 mm in this study, which resulted in a low false positive rate (5/200).

CXA, also called the clivocanal angle, reflects the angle between the clivus plane and C2 vertebrae, ranging from 150° in flexion and 180° in extension in a normal adult population [6]. In our present study, CXA ranged from 130° to 172° in the control group and using the traditional criterion (150°) would result in a very high false positive rate (36.5%, 73/200 cases) [6]. This variation might reflect the confounding effect caused by the subjects’ cervical curvature and hyperostosis [6, 12]. This might also be partly due to the newer method we used to measure CXA. The Wackenheim line used in this study ran along the lower third of the clivus, whereas traditionally the line extends from the top of the dorsum sellae to the basion (Fig. 2d) [12, 13, 17]. The newer method was recently recommended to standardize the measurement method for CVJ evaluation, which can be used to analyze both neck and head MRI data [6]. Many patients with BI complain of symptoms similar to those of cervical radiculopathy and undergo cervical MRI examination clinically. In the present data, most cases were cervical spine images; thus, the clivus was not completely covered and traditional measurement of CXA was impossible.

CDA was a new BI indicator proposed by Xu and Gong [13], who claimed that it could evade the influence from the posterior surfaces of the clivus and the axis that are not perfectly flat planes. However, CDA showed lower AUC than CXA in the present study, which was not in agreement with the previously reported [10]. Moreover, we found that the deformities or morphological variants of the odontoid process were rather common, especially in patients with BI, which may have an adverse effect on accurate measurement of CDA, as supported by the lowest inter-observer agreement in the present study.

The CPA proposed in this study is a naturally existing angle formed by the hard palate line and the Wackenheim clivus line. Interestingly, although the hard palate line has been used in the Bull angle and modified Omega angle and the Wackenheim line has been widely used to evaluate CVJ abnormalities, the CPA, formed at the intersection of these two lines, has not been proposed for use in this regard before [9, 15, 18–21]. Our study demonstrated that CPA could accurately reflect the changes of craniovertebral angle. Of note, the AUC of CPA in diagnosing BI was similar to that of CXA or CDA. Furthermore, CPA exhibited the following peculiar advantages. First, both the hard palate line and Wackenheim line are clearly depicted as a fine line on midsagittal MRI T1-weighted image (T1WI) or T2-weighted image (T2WI), which facilitates identification and measurement and thus leads to higher inter-observer agreement. Second, congenital malformations (e.g., os odontoideum), fracture or dislocation of the dens, osteosclerosis of the posterior margin of the axis, and ossification of ligaments can affect measurements of CXA and CDA [8, 10]. Also, CXA or CDA was affected by the curvature of cervical spine [6, 11]. In contrast, the osseous basis of the hard palate is the palatine process of maxilla and the horizontal plate of the palatine bone, which are stable and unaffected by the aforementioned factors. Moreover, facial hypoplasia or hard palate abnormality is rare and will not affect the measurements [19]. In short, CPA exhibited less variation when compared with CXA and CDA.

Moreover, any single parameter varies within a normal range, which necessitates assessing at least two different measures for patients with suspected BI [12]. The present study demonstrated that sensitivity, specificity, and accuracy in diagnosing BI were significantly improved when CPA + CDA or CPA + CXA was used. The mechanism underlying this phenomenon is unclear. CPA may reflect BI differently from CDA or CXA. CPA seems to mainly reflect the degree of clivus tilt toward the horizontal plane because the hard palate is stable and unaffected by the relative movement of the head and the cervical spine in the setting of BI [19]. In contrast, CXA (or CDA) is influenced by a great variety of accompanied anomalies affecting this region, such as C2–3 assimilation, atlas-axis subluxation, and atlas occipitalization [6, 11, 13, 22].

Our study has several limitations. This is a retrospective study based on MRI, where CT data were not available in many cases. This may affect the measurement of CL, CDA, and CXA because MRI is not the best choice to depict bone structure [23]. However, we identified bone cortex as best as we could and our results are highly comparable to other published reports [13, 17]. Further, this study did not examine the relations of CPA with other indicators (e.g., McRae line, Klaus index, and Welcher basal angle) or with clinical manifestations, which shall be addressed in future studies [11, 20]. In addition, future studies should address the value of CPA in evaluating treatment efficacy of BI.

In conclusion, on midsagittal MRI T1WI or T2WI of the head or neck, CPA accurately reflected the changes of craniovertebral angle with higher inter-observer agreement than that of CXA or CDA. Patients with BI had a sharper CPA compared to control subjects. CPA showed similar diagnostic performance with CXA or CDA with the cutoff value being 53.5° and provided additional diagnostic value to CXA and CDA for BI. Therefore, CPA can serve as a useful sentinel to alert the radiologist and surgeon to the possibility of CVJ deformity.

## Electronic supplementary material

ESM 1 (DOCX 131 kb)

## Abbreviations

BI: Basilar invagination
CDA: Clivodens angle
CI: Confidence interval
CL: Chamberlain line
CPA: Clivopalate angle
CVJ: Craniocervical junction
CXA: Clivoaxial angle
