# Supplementary material for: Clivopalate angle: a new diagnostic method for basilar invagination at magnetic resonance imaging
Source: Eur Radiol. 2019 Feb 8;29(7):3450–7. doi: 10.1007/s00330-018-5972-3 (PMC6554245; doi:10.1007/s00330-018-5972-3)
Supplement: Supplementary file 1 — (DOCX 131 kb) [file 330_2018_5972_MOESM1_ESM.docx]

**Supplementary Tables**

| **Supplementary Table 1.** The sequences and parameters of head or neck MRI scans | | | | | | | | | |
| --- | --- | --- | --- | --- | --- | --- | --- | --- | --- |
|  | Sequence |  | Philips, Achieva | | |  | GE, Signa Excite | | |
|  |  | TR/TE (ms) | FOV  (mm) | Matrix | Slice  (mm) | TR/TE  (ms) | FOV  (mm) | Matrix | Slice  (mm) |
| Head | Sagittal T1WI | 2000/20 | 100 | 328 × 223 | 5 | 1970/23 | 100 | 384 × 224 | 5 |
|  | Axial T2WI | 3000/80 | 180 | 384 × 256 | 6 | 4640/99 | 180 | 420 × 306 | 6 |
| Neck | Sagittal T1WI | 581/8 | 156 | 270 × 228 | 3 | 2075/27 | 100 | 320 × 192 | 3 |
|  | Sagittal T2WI | 2061/143 | 156 | 288 × 224 | 3 | 3420/116 | 100 | 308 × 140 | 3 |
|  | Sagittal T2WI FS | 3038/81 | 156 | 356 × 132 | 3 | 3450/42 | 100 | 288 × 192 | 3 |

TR time of repetition, TE time of echo, FOV field of view, T1WI T1-weighted imaging, T2WI T2-weighted imaging, FS fat suppression.

| **Supplementary Table 2.** Comparison of the AUC values of angles individually and in combinations | | | | | |
| --- | --- | --- | --- | --- | --- |
| Parameter | AUC Difference | 95% CI | Z value | P value |  |
| CPA vs CXA | 0.020 | -0.009-0.048 | 1.332 | 0.183 |  |
| CPA vs CDA | 0.013 | -0.025-0.050 | 0.659 | 0.510 |  |
| CXA vs CDA | 0.032 | 0.013-0.051 | 3.371 | <0.001 |  |
| CXA+CDA vs. CXA | 0 | -0.011-0.12 | 0.632 | 0.681 |  |
| CPA+CXA vs. CXA | 0.016 | 0.009-0.023 | 2.221 | 0.022 |  |
| CPA+CDA vs. CDA | 0.039 | 0.022-0.056 | 3.311 | 0.007 |  |
| CPA+CXA vs CPA+CDA | 0.009 | 0.001-0.017 | 2.076 | 0.038 |  |
| CPA+CXA vs CXA+CDA | 0.016 | 0.002-0.030 | 2.194 | 0.028 |  |
| CPA+CDA vs CXA+CDA | 0.007 | -0.009-0.024 | 0.875 | 0.381 |  |

AUC area under the curve, CI confidence interval, CPA clivopalate angle, CXA clivoaxial angle, CDA clivodens angle.

**Supplementary Figure**


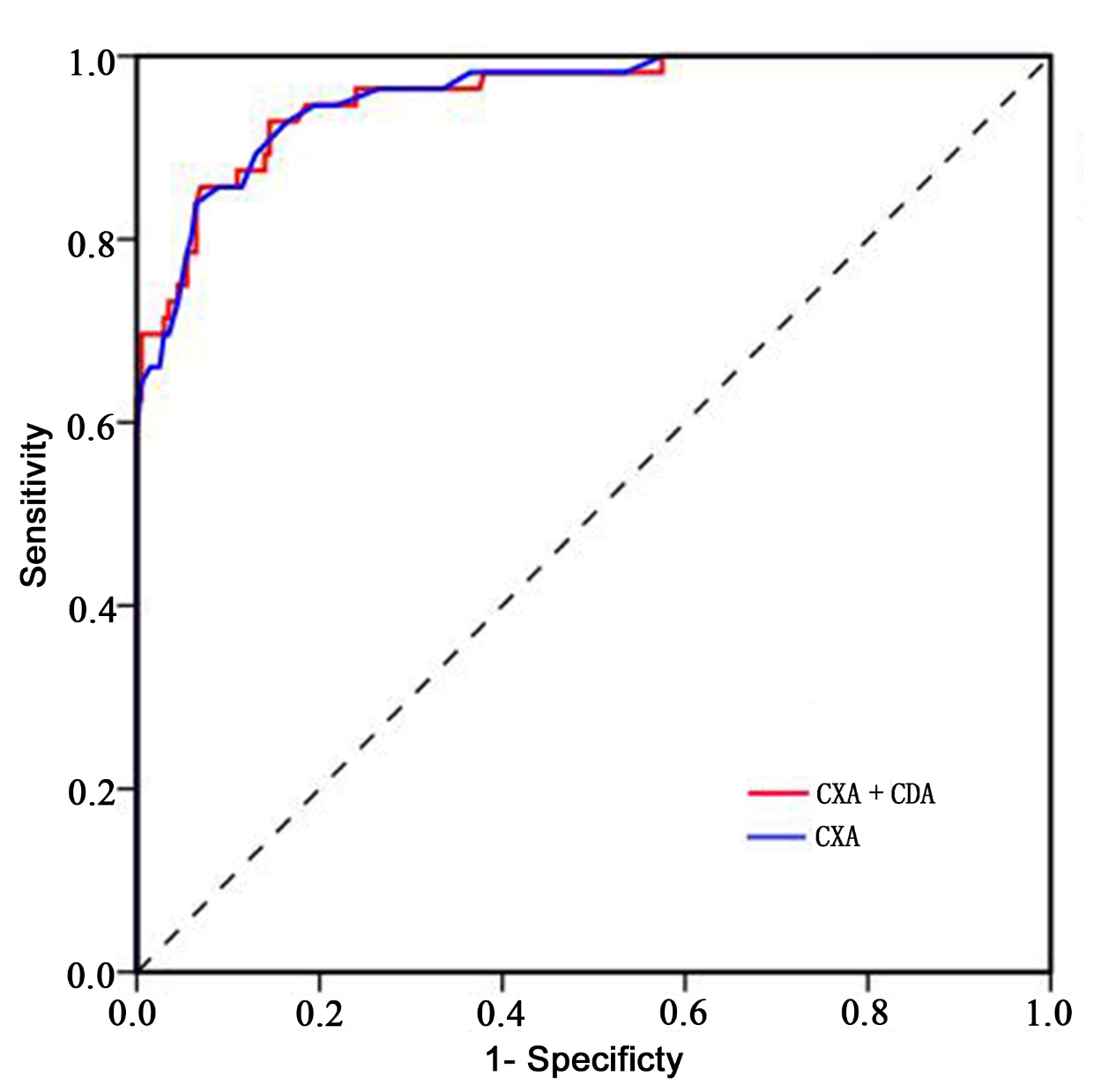


**Supplementary Fig 1.** Receiver operating characteristic (ROC) curve of clivoaxial (CXA) and the combination of CXA and clivodense (CDA). The combination of CXA and CDA does not improve the diagnostic performance beyond that of CXA.
